# Nano-Modified Polymer Gels as Temperature- and Salt-Resistant Fluid-Loss Additive for Water-Based Drilling Fluids

**DOI:** 10.3390/gels8090547

**Published:** 2022-08-29

**Authors:** Jian Li, Jinsheng Sun, Kaihe Lv, Yuxi Ji, Jintao Ji, Jingping Liu

**Affiliations:** 1 School of Petroleum Engineering, China University of Petroleum (East China), Qingdao 266580, China; 2 CNPC Engineering Technology R&D Company Limited, Beijing 102206, China; 3 Inspection and Testing Center of Huabei Oil Field Company, PetroChina, Renqiu 062552, China; 4 Petroleum Machinery Plant of Bohai Petroleum Equipment Manufacturing Company, PetroChina, Renqiu 062552, China

**Keywords:** nano-modified polymers gels, fluid-loss additive, temperature- and salt-resistant, water-based drilling fluids, ultra-deep wells

## Abstract

With the continuous exploration and development of oil and gas resources to deep formations, the key treatment agents of water-based drilling fluids face severe challenges from high temperatures and salinity, and the development of high temperature and salt resistance filtration reducers has always been the focus of research in the field of oilfield chemistry. In this study, a nano-silica-modified co-polymer (NS-ANAD) gel was synthesized by using acrylamide, isopropylacrylamide, 2-acrylamide-2-methyl propane sulfonic acid, diallyl dimethyl ammonium chloride, and double-bond-modified inorganic silica particles (KH570-SiO_2_) through free radical co-polymerization. The introduction of nanotechnology enhances the polymer’s resistance to high temperature degradation, making it useful as a high-temperature-resistant fluid loss reducer. Moreover, the anions (sulfonates) and cations (quaternary ammonium) enhance the extension of the polymer and the adsorption on the surface of bentonite particles in a saline environment, which in turn improves the salt resistance of the polymer. The drilling fluids containing 2.0 wt% NS-ANAD co-polymer gels still show excellent rheological and filtration performance, even after aging in high temperature (200 °C) and high salinity (saturated salt) environments, showing great potential for application in deep and ultra-deep drilling engineering.

## 1. Introduction

Drilling fluids, known as the blood of drilling, are essential for stabilizing the wellbore, carrying cuttings, and cooling and lubricating the drillbit during the drilling process [1,2,3]. Generally, drilling fluids can be divided into water-based drilling fluids (WBDFs), oil-based drilling fluids (OBDFs), and foam drilling fluids, according to their composition [4]. WBDFs, a colloidal dispersion composed of water, bentonite, and various chemical additives, are widely used in drilling engineering due to their relatively economical and environmentally-friendly advantages [5,6]. However, with the rapid growth of global demand for energy resources and the depletion of conventional shallow oil/gas resources, global oil exploration and development is gradually advancing from medium-shallow to deep, ultra-deep, and other unconventional oil/gas resources. The current WBDFs are not suitable for the complex geological characteristics of deep and ultra-deep wells (such as high temperature, high pressure, high stress, high salt content, etc.) [7,8]. Therefore, developing anti-high-temperature and –high-salt water-based drilling fluid technology is of great significance for the safe, economic, and efficient drilling of deep and ultra-deep wells.

Fluid loss reducer, as one of the most essential additives in WBDFs, which can fully control the rheology and filtration performance of drilling fluids, has attracted extensive attention from petroleum engineering researchers in academia and industry [9,10,11,12]. The common fluid loss reducers are mainly divided into two categories: natural/natural-modified polymers and synthetic polymers, which can optimize the particle size distribution of bentonite particles and reduce the permeability of mud cakes, thereby achieving the effect of reducing drilling fluid filtration [13]. Natural fluid loss additives, including humic acid, cellulose, lignin, starch, phenolic resin, etc., have been widely used in conventional formation drilling operations due to their vast sources and lack of environmental harm [14,15,16,17,18,19,20]. Nevertheless, due to a large number of ether–oxygen bonds (low bond energy) in the natural polymer molecular structure, its temperature resistance is poor (<150 °C), limiting its application in deep and ultra-deep wells. In addition, the current natural fluid loss reducer is added in large amounts (>5 wt%), which will have a negative impact on the control of the rheological property of the drilling fluid system and even reduce the drilling rate. Synthetic polymer fluid loss reducers, which refers to co-polymers formed by free radical polymerization of vinyl functional group-containing monomers, can be adjusted through functional monomers with different adsorption groups, hydration groups, and anti-salt groups, thus improving the thermal stability and salt resistance of the co-polymer. A series of synthetic polymers based on monomers, such as acrylamide (AM), 2-acrylamide-2-methyl propane sulfonic acid (AMPS), and N-vinylpyrrolidone (NVP) have been used in the field of drilling fluids as anti-high-temperature fluid loss additives [21,22,23,24]. For example, the co-polymers with acrylamide as a hydrophilic monomer and styrene as a hydrophobic monomer were synthesized via inverse emulsion terpolymerization, which shows excellent temperature and salt resistance [25,26]. Despite the sufficient development of synthetic polymer fluid loss control agents, the anti-degradation and anti-curling capabilities of polymers in ultra-deep wells, where there is both high temperature (>200 °C) and high salinity (>100,000 mg/L), still face significant challenges.

In recent years, nanomaterials with unique physical and chemical properties exhibited extraordinary performance in drilling fluid systems like high-temperature stability, remarkable rheological properties, and filtration properties, pointing out a new direction for high-temperature resistant drilling fluid. Various nanomaterials, including inorganic nanomaterials (such as graphene oxide, silica, laponite, carbon nanotubes, calcium carbonate, etc.), organic nanomaterials (such as nanospheres, nano-gels, nano-emulsions, etc.), and organic/inorganic composite nanomaterials, have been added into drilling fluids as filtrate reducers [27,28,29,30,31,32]. Furthermore, nanocomposites prepared by combining inorganic nanomaterials and polymers are especially beneficial for enhancing the dispersibility of nanomaterials and improving the temperature and salt resistance of polymers. For example, a nano-silica graft co-polymer was prepared by inverse emulsion polymerization, which exhibited an excellent filtration performance at high Na^+^/Ca^2+^ concentrations [33]. Bai et al. grafted N-isopropyl acrylamide (NIPAM) onto the surface of vinyltrimethoxy-modified SiO_2_ to synthesize a thermosensitive polymer plugging agent, showing excellent high-temperature resistance and plugging performance [34]. Therefore, considering the thermal stability of inorganic nanoparticles and the high performance of some polymers, we believe that obtaining high-temperature and high-salt fluid loss reducers is feasible.

**Figure 1:**
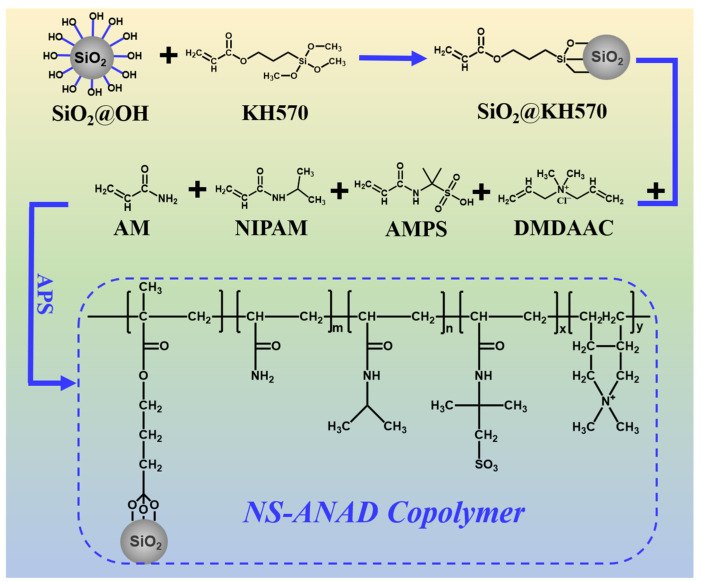
The synthesis procedure and structural formula of NS-ANAD co-polymer.

In this paper, the nano-silica-modified co-polymer gel was developed as a high-temperature- and salt-resistant fluid loss reducer. As shown in Figure 1, the inorganic/organic polymer fluid loss reducer (i.e., NS-ANAD) was synthesized by using acrylamide (AM), isopropylacrylamide (NIPAM), 2-acrylamide-2-methyl propane sulfonic acid (AMPS), diallyl dimethyl ammonium chloride (DMDAAC), and double-bond-modified inorganic silica particles (KH570-SiO_2_) through free radical co-polymerization. The introduction of nanotechnology limits the mobility of polymer chains in high temperature environments, further improving the temperature resistance of polymers. In addition, the sulfonic acid group with excellent salt resistance enhances the stretching performance of the polymer in the salt water environment and improves the salt resistance of the polymer. Moreover, the cationic groups enhanced the adsorption of polymer chains on the surface of bentonite particles and promoted the hydration and dispersion of bentonite particles. Therefore, after elegant monomer ratio optimization, the NS-ANAD-based drilling fluids still show excellent rheological and filtration performance even after aging in high temperatures (up to 200 °C) and high salinity (saturated salt) environments.

## 2. Results and Discussion

### 2.1. Characterization of NS-ANAD

**Figure 2:**
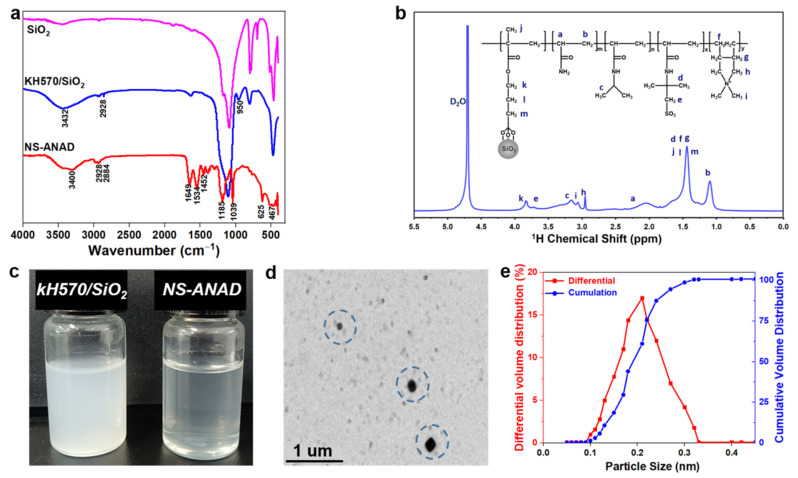
(**a**) FTIR of SiO_2_, KH570/SiO_2_, and NS-ANAD co-polymers. (**b**) ^1^H NMR curves of NS-ANAD co-polymer. (**c**) 2 wt% aqueous solution of KH570/SiO_2_ and NS-ANAD co-polymer. (**d**) TEM image of NS-ANAD co-polymer. (**e**) Particle size distribution of NS-ANAD co-polymer.

Firstly, the chemical structure of the NS-ANAD co-polymer was characterized by Fourier transform infrared spectroscopy (FT-IR) and nuclear magnetic resonance spectroscopy (^1^H-NMR). As shown in Figure 2a, the peak at 2928 cm^−1^ was the vibration absorption peak of the methyl group in KH570, and the peak at 950 cm^−1^ was the vibration absorption peak of the double bond in KH570, indicating that the silane coupling agent, KH570, had been successfully grafted on the surface of nano-SiO_2_. From the FT-IR of the NS-ANAD co-polymer, it could be seen that the peak corresponding to 3400 cm^−1^ was the stretching vibration absorption peak of -NH, and the peak corresponding to 1649 cm^−1^ was the stretching vibration absorption peak of C=O, indicating that monomer AM and NIPAM participated in the polymerization reaction. The peak at 625 cm^−1^ corresponds to the stretching vibration absorption peak of -CS in AMPS, while the peaks at 1039 cm^−1^ were attributed to the stretching vibrations of the sulfonic acid (−SO_3_) groups in AMPS. The peak corresponding to 1534 cm^−1^ was the vibration absorption peak of −CN in the five-membered ring, confirming the polymerization of monomer DMDAAC. In addition, the characteristic peaks of double bonds were not shown in the infrared spectrum of the NS-ANAD co-polymer, which proved that all monomers were fully reacted. Moreover, the chemical shifts of some protons in the NS-ANAD were given in the ^1^H NMR spectra, and the corresponding peaks were marked. The results showed that AM, NIPAM, AMPS, and DMDAAC were successfully co-polymerized with KH570/SiO_2_, which further indicated that the molecular structure of the NS-ANAD co-polymer was consistent with the design.

It can be seen from Figure 2c that the aqueous solution of KH570/SiO_2_ was relatively turbid, and the solution will settle after a period of time (picture not given), indicating that the hydrophilicity of KH570/SiO_2_ is poor. However, the solution of NS-ANAD became clearer due to the hydrophilic groups (such as −SO_3_^−^ and −NH_2_) grafted on the surface of the SiO_2_ particles, which increased the hydrophilicity of the nanoparticles, further enhancing its dispersibility in water. In addition, a large number of uniformly-dispersed single spherical nanoparticles with diameters of 200 nm were observed in the TEM images, further indicating that the grafting of the polymer promotes the dispersibility of the nanoparticles. Moreover, as shown in Figure 2e, the particle size distribution of the NS-ANAD was between 100 and 320 nm, with a median particle size (D50 value) of 200 nm, which was consistent with the TEM results.

**Figure 3:**
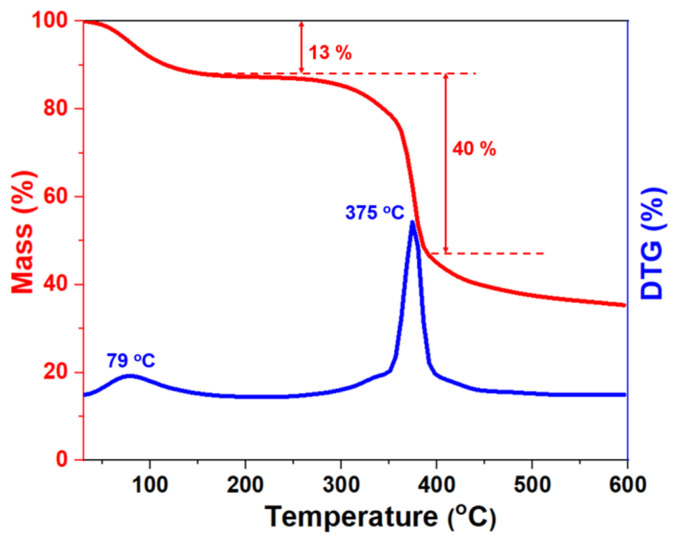
TGA and DTG curves of NS-ANAD co-polymer.

Generally speaking, the formation temperature would increase by 2–3 °C for every 100 m of drilling depth. Currently, the deepest reservoir detected was nearly 9 km with a bottom hole temperature of 180–260 °C. Therefore, the tolerance of drilling fluids to ultra-high temperatures has been a major challenge in deep and ultra-deep wells. To demonstrate the thermal stability of the NS-ANAD co-polymer, thermogravimetric analysis (TGA) and differential thermogravimetric (DTG) curves of the NS-ANAD co-polymer were performed in an N_2_ atmosphere, with three stages of thermal weight loss (Figure 3). The first stage ranged from 40–255 °C, and the peak occurred at 73 °C, with a mass loss of 13%, which was caused by the volatilization of a small amount of adsorbed water on the fluid loss reducer. In the second stage, with the temperature ranging from 280–438 °C and the peak occurring at 375 °C, the mass loss was about 40%, which mainly corresponded to the decomposition of the amide group, sulfonic groups, and C-C bonds in the main chains of the NS-ANAD co-polymer. When the temperature exceeded 440 °C, the NS-ANAD co-polymer gradually carbonized, thus causing mass loss. Due to the high density of double bonds on the surface of inorganic nano-silica and the high specific surface area, a small amount of KH570/SiO_2_ will increase the cross-linking points of the polymer network, thereby limiting the chain segment mobility of the polymer and increasing the temperature resistance of the polymer. Therefore, the TGA experiments show that the NS-ANAD co-polymer has excellent thermal stability and does not undergo thermal decomposition below 280 °C, showing great potential for application in deep and ultra-deep well drilling operations.

### 2.2. Performance of NS-ANAD in WDFs

**Figure 4:**
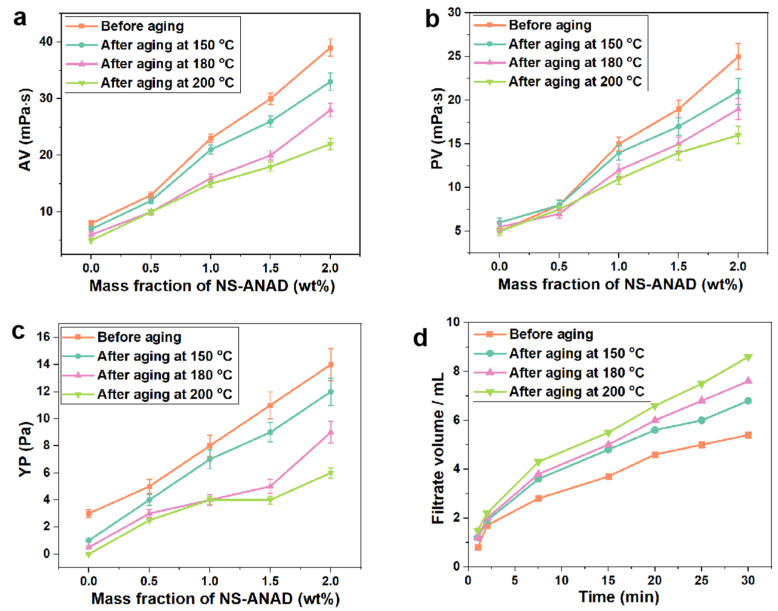
Rheological properties of drilling fluid with different contents of NS-ANAD co-polymer before and after aging at different temperatures for 16 h: (**a**) apparent viscosity (AV); (**b**) plastic viscosity (PV); (**c**) yield point (YP). (**d**) Fluid loss volume (FL_API_) of drilling fluid with 2 wt% NS-ANAD before and after aging at different temperatures for 16 h.

Given that the rheological and fluid loss properties of the drilling fluids are significant for carrying cuttings and maintaining the stability of the wellbore during the drilling process, the rheological parameters (AV, PV, YP) and fluid loss (FL_API_) of drilling fluids with different contents of NS-ANAD co-polymers were compared at a room temperature of 25 °C and high temperature of 150 °C, 180 °C, and 200 °C. As shown in Figure 4a–c, with the increase of NS-ANAD, the rheological parameters of drilling fluids all increased. When the concentration of NS-ANAD was 2.0 wt%, the AV, PV, and YP values of the system before aging were about 39 mPa·s, 25 mPa·s, and 14 Pa, respectively. The high temperatures will aggravate the thermal movement of bentonite particles in the drilling fluid system, resulting in their aggregation and flocculation. At the same time, high temperatures would cause the degradation of the polymer molecular chain and the cross-linking reaction of some functional groups. Thus, the interaction between polymer and bentonite particles would be weakened at high temperatures, thus maintaining the stability of the drilling fluid system was a considerable challenge. As can be seen from Figure 4a–c, the rheological parameters of the drilling fluids system decreased to a certain extent in the high temperature environment, where the AV, PV, and YP of the drilling fluids with 2 wt% NS-ANAD after aging at 200 °C decreased to 20 mPa·s, 15 mPa·s, and 5 Pa, respectively. Fortunately, it could be seen from Figure 4d that the fluid loss volume of the drilling fluids system after high temperature aging did not show a sharp increase trend, and the fluid loss volume of the drilling fluids containing 2 wt% NS-ANAD after aging at 200 °C was only just 8 mL. This was mainly attributed to the excellent temperature resistance, adsorption, and hydration properties of the NS-ANAD co-polymer, which improved the stability of the drilling fluids in a high temperature environment, thereby reducing the filtration loss.

**Figure 5:**
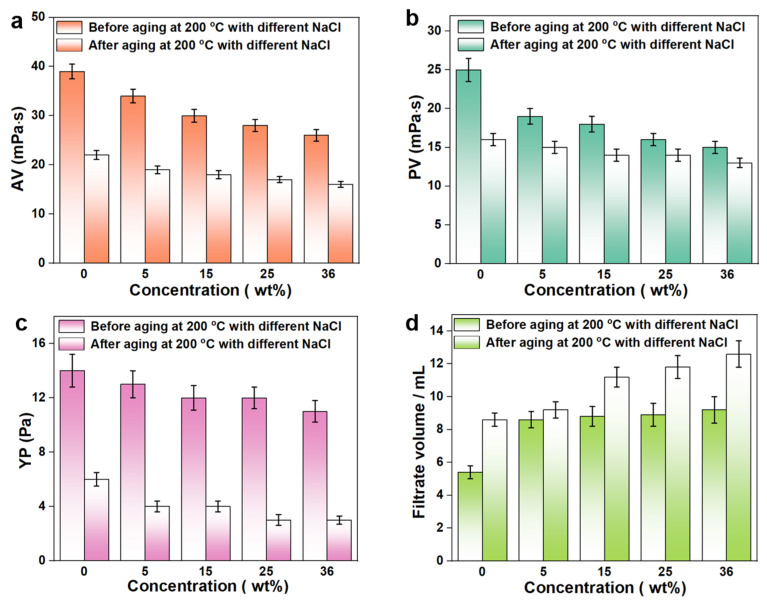
Rheological and fluid loss properties of 2 wt% NS-ANAD-based drilling fluids with different fractions of NaCl before and after aging at 200 °C for 16 h: (**a**) apparent viscosity (AV); (**b**) plastic viscosity (PV); (**c**) yield point (YP); (**d**) fluid loss volume (FL_API_).

Salt-gypsum layers often appear in deep formations, where a large amount of sodium chloride (NaCl) and other inorganic salts would invade into the drilling fluids from the formation, causing a negative impact on the performance of the drilling fluids. For example, metal cations would destroy the electrostatic repulsion between bentonite particles, leading to the aggregation or flocculation of bentonite and weakening the hydration and dispersing ability of bentonite particles. In addition, the increased metal counter-ions in the drilling fluids gradually approach the polyelectrolyte segment, which would cause the negative charges on the segment to be shielded, resulting in the curling of the polymer chain and the reduction of the hydrodynamic volume. Usually, engineers take the method of adding NaCl to the drilling fluids (the concentration can reach saturation) to prepare the brine drilling fluids so that the drilling fluids system has a stronger salt resistance, thereby alleviating the effect of salt in the salt-gypsum layer on the drilling fluids. To evaluate the anti-salt capability of NS-ANAD-based drilling fluids, the rheological and fluid loss properties of the base slurry containing 2 wt% NS-ANAD corresponding to a series of NaCl concentrations were studied. As can be seen from Figure 5, the AV of the drilling fluids changed from 39 mPa·s to 26 mPa·s, the PV changed from 25 mPa·s to 15 mPa·s, the YP changed from 14 Pa to 11 Pa, and the FL_API_ increased from 5.2 to 9.2 mL after contamination with 36 wt% NaCl, which confirmed that salt can have a serious negative impact on the rheology of the drilling fluids. However, the rheology and fluid loss of the drilling fluids after aging in a high temperature (200 °C) and high salt (saturated salt) environment still maintained a certain ideal value range (~12 mL), which demonstrated that the NS-ANAD co-polymer had a certain temperature resistance and salt resistance, indicating its potential application in deep and ultra-deep well drilling.

### 2.3. Filtration Control Mechanism Analysis

**Figure 6:**
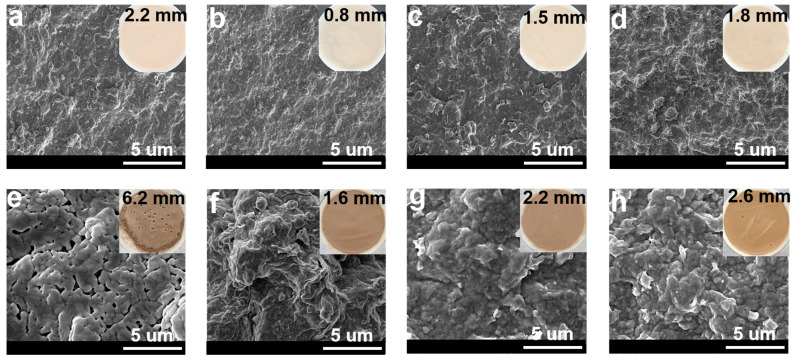
SEM and digital images of mud cakes deposited from different drilling fluids: (**a**,**e**) 4% bentonite, (**b**,**f**) 4% bentonite + 2.0 wt% NS-ANAD, (**c**,**g**) 4% bentonite + 2 wt% NS-ANAD + 5 wt% NaCl, (**d**,**h**) 4% bentonite + 2 wt% NS-ANAD + 36 wt% NaCl. (**a**–**d**) Mud cake formed at 25 °C. (**e**–**h**) Mud cake formed after aged at 200 °C for 16 h.

During the drilling process, to prevent the occurrence of blowout accidents, the liquid column pressure of the drilling fluids was slightly greater than the formation pressure, so the solid phase particles in the drilling fluids would be deposited on the wellbore to form a layer of mud cake. The quality of the mud cake not only directly affects the fluid loss of the drilling fluids, but also has a close relationship with the wellbore stability and reservoir protection. Generally speaking, the appearance of a good mud cake should be dense and compact, with few leakage pores, to prevent the filtrate from percolating into the formation. Therefore, to analyze the mechanism of the NS-ANAD co-polymer, it is necessary to study its influence on the morphology of the mud cake. As shown in Figure 6a, the base slurry formed a relatively smooth, uniform, and dense mud cake with a thickness of about 2.2 mm. However, after aging at 200 °C for 16 h, the thickness of the mud cake increased by 6.2 mm, and pores were observed on the surface of the mud cake (Figure 6e), which was due to the high temperature that aggravated the aggregation of bentonite particles, thus resulting in the poor quality of the mud cake. When 2 wt% NS-ANAD co-polymer was added to the base slurry, a thin and tough mud cake was formed with a thickness of about 0.5 mm (Figure 6b), which confirmed that the NS-ANAD co-polymer could improve the quality of mud cake. Moreover, even after aging at 200 °C for 16 h, the thickness of the mud cake was still controllable, and the stacked structure of the bentonite particles was relatively tight, with no apparent pores and micro-cracks (Figure 6f). This result shows that the NS-ANAD co-polymer still had strong adsorption capacity on bentonite particles after aging at high temperature, which effectively inhibited the coalescence of bentonite particles, thus improving the temperature resistance of drilling fluids and the quality of the mud cake.

As shown in Figure 6c,g, when 5 wt% sodium chloride was added to the drilling fluids containing 2 wt% NS-ANAD, the thickness of the mud cake formed by the drilling fluids increased to 1.5 mm, and some large bentonite particles were attached to the surface of the mud cake. This was because the metallic sodium ions compressed the electric double layer and reduced the repulsion between the bentonite particles, resulting in the aggregation of the bentonite particles, which in turn increased the roughness of the mud cake. In addition, as can be seen from Figure 5d, with a further increase in the sodium chloride concentration (saturated salt), the thickness of the mud cake also increased slightly. However, the particle size distribution of the bentonite particles was still relatively suitable, and the packing between the particles was rather tight. Even after aging in a high temperature (200 °C), high salt (saturated salt) environment, the mud cake formed by the drilling fluid did not show obvious pores and micro-fractures (Figure 6h), indicating that the NS-ANAD co-polymer still had a good adsorption capacity and maintained an ideal particle size distribution of bentonite particles in a high temperature and high salt environment, thus showing its application potential in deep wells and ultra-deep wells.

**Figure 7:**
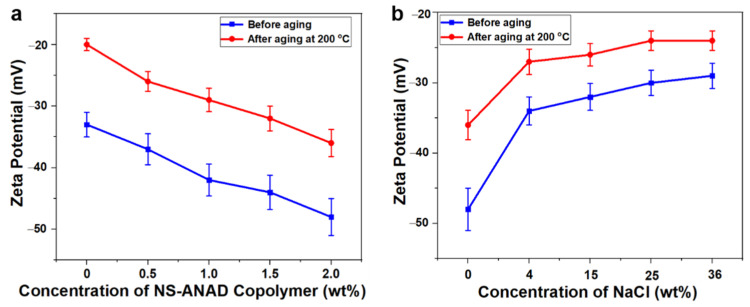
(**a**) Zeta potential curves of NS-ANAD-based drilling fluids before and after aging at 200 °C. (**b**) Zeta potential curves of 2 wt% NS-ANAD co-polymer-based drilling fluids with different fractions of NaCl before and after aging at 200 °C.

Drilling fluid is a colloidal dispersion system formed by bentonite particles, chemical treatment agents, and water, thus the effect of the NS-ANAD co-polymer on the colloidal stability of drilling fluids can be revealed through the analysis of Zeta potential. According to the DLVO theory, the Zeta potential can accurately reflect the electrostatic repulsion between bentonite particles, which in turn reflects the colloidal stability of the drilling fluids. Generally speaking, if the absolute value of the Zeta potential of the drilling fluids is greater than 30 mV, it can be considered that the drilling fluid colloid is stable and the particle size distribution is reasonable. As shown in Figure 7a, the Zeta potential value of the drilling fluid base slurry is about −34 mV, which decreases to about −20 mV after hot rolling at 200 °C for 16 h. This was because the high temperature compressed the diffusion electric double layer between the bentonite particles, which destroyed the hydration and dispersibility of the bentonite, thus further reducing the stability of the drilling fluids. However, with the increase of the NS-ANAD co-polymer, the Zeta potential (absolute value) of the drilling fluids before and after aging gradually increased, indicating that the addition of NS-ANAD helps to increase the stability of the drilling fluids. The fundamental reason was that the NS-ANAD co-polymer can be adsorbed on the surface of bentonite particles, and the hydration groups, such as sulfonic acids and amides, on the polymer molecular chain increased the thickness of the hydration film on the bentonite particles, thereby increasing the Zeta potential of the drilling fluids. When drilling into the salt-gypsum layer, the drilling fluid system would face the invasion of high concentrations of metal cations, which would compress the diffusion electric double layer of the bentonite particles, thereby reducing the Zeta potential of the drilling fluids. Fortunately, as shown in Figure 7b, although the Zeta potential value of the drilling fluid containing 2 wt% NS-ANAD gradually decreased with the increase of salt concentration, the drilling fluid still had an ideal Zeta potential after aging at 200 °C and a saturated salt environment, indicating that NS-ANAD could still effectively maintain the uniform dispersion of bentonite particles in a high temperature and high salinity environment.

### 2.4. Potential Mechanism Analysis

**Figure 8:**
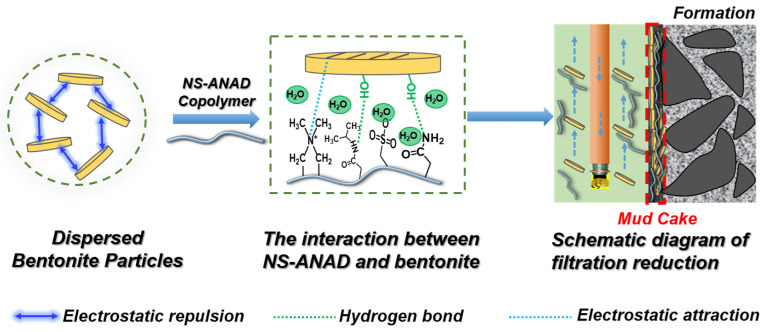
Schematic illustration on the mechanism of NS-ANAD co-polymer in enhancing the rheology and filtration performance of drilling fluid.

Usually, the surface of bentonite is negatively charged due to the ion exchange in the lattice of clay minerals, which causes a huge electrostatic repulsion between the bentonite particles, thus promoting the dispersion of the bentonite particles [14,15]. When the NS-ANAD copolymer was added to the base slurry, the polymer would be firmly adsorbed on the surface of the bentonite particles due to the electrostatic attraction formed between the negative charge on the surface of the bentonite and the cationic groups in the polymer [16]. In addition, the hydrogen bonding force would also promote the entanglement of polymer chains on the surface of the bentonite particles, thereby forming a protective polymer layer on the surface of the bentonite. The sulfonic acid group with excellent salt resistance would enhance the anti-crimping ability of the polymer in a salt water environment, which enhanced the ability of the polymer to control the rheological properties [27,28,29]. Moreover, the cations on the polymer could also shield the negative effects of metal cations on the sulfonate, thereby enhancing the polymer’s hydration and fluid loss reduction capabilities. Therefore, as shown in Figure 8, once the NS-ANAD co-polymer was added to the mud system, it would hydrate into elastic coils of different conformations with a particular hydrodynamic volume. The polar groups and ionic groups of the polymer molecular chain were adsorbed on the surface of bentonite minerals by hydrogen bonds and coulomb attraction force, forming a layered structure of “bentonite-molecular chain-water molecules”. Under the action of the positive pressure difference between the drilling fluid column and the formation, this layered structure would accumulate on the surface of the wellbore to form a layer of mud cake. Due to the high water content and good deformability of the layered structure, it could fill the gap of the mud cake with an irregular shape after entering the hole, thereby reducing the porosity and permeability of the mud cake and reducing the filtration of the drilling fluid.

### 2.5. Comparison with Other Filtrate Reducers

**Figure 9:**
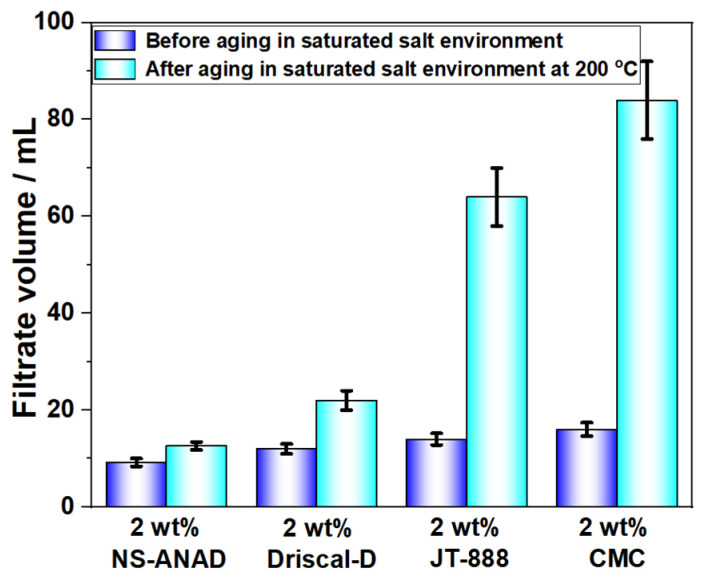
Comparison of NS-ANAD co-polymer and different commercial fluid loss control agents.

The synthesized co-polymer was compared with the zwitterionic polymer (JT-888), carboxymethyl cellulose (CMC) and Driscal-D, which were commonly used filtrate reducers in the drilling industry. As shown in Figure 9, the synthetic fluid loss reducer, NS-ANAD, had a significantly better fluid loss reduction effect than JT-888, CMC, and Driscal-D, indicating that NS-ANAD has a good fluid loss reduction effect in a high temperature and high salt environment. However, since some of the monomers (NIPAM) used in the synthesis of NS-ANAD are relatively expensive, their cost was higher than that of JT-888 and CMC, and may be comparable to that of Driscal-D. Therefore, in order to make the product go to the application market, we should deeply study the regulation of the monomer ratio to make the prepared polymer cost-effective.

## 3. Conclusions

In summary, a nano-modified polymer gel (NS-ANAD) composed of KH570/SiO_2_, AM, NIPAM, AMPS, and DMDAAC was synthesized by aqueous free-radical polymerization as a novel anti-high-temperature and anti-salt contamination filtrate reducer. The chemical molecular structure of NS-ANAD has been characterized by FTIR and ^1^H-NMR, which confirmed that all of the monomers participated in the polymerization reaction. As TG analysis shows, the NS-ANAD has excellent thermal stability, and the degradation temperature of the co-polymer was above 280 °C, demonstrating its potential for application in high-temperature-resistant drilling fluids. The rheological and filtration loss properties evaluation results demonstrated that a base slurry with 2.0 wt% NS-ANAD could resist saturated salt (36 wt% NaCl) contamination and aging at 200 °C. Moreover, the mechanism analysis shows that the NS-ANAD could be firmly adsorbed on the surface of bentonite particles through electrostatic attraction and hydrogen bonding, forming a polymer protective layer to control the particle size distribution of the drilling fluid within an ideal range. Thus, a thin, dense, and tough mud cake can be formed on the wellbore, thus reducing water loss. Therefore, the excellent rheological and filtration control performance of NS-ANAD in ultra-high temperature and high salinity conditions shows its great potential for application in deep or ultra-deep drilling engineering.

## 4. Materials and Methods

### 4.1. Materials

Acrylamide (AM, 98 wt%), 2-acrylamide-2methyl propane sulfonic acid (AMPS, 98 wt%), isopropylacrylamide (NIPAM, 98 wt%), diallyl dimethyl ammonium chloride (DMDAAC, 98 wt%), and γ-Methacryloxypropyltrimethoxysilane (KH570) were purchased from Aladdin Reagent Corporation (Shanghai, China). Nano-silica (SiO_2_) was purchased from Xianfeng Nano Corporation (Nanjing, China). Initiator ammonium persulfate (APS, 99 wt%) was obtained from Sinopharm Chemical Reagent Co., Ltd. (Shanghai, China). Sodium hydroxide was obtained from Macklin Biochemical Corporation (Shanghai, China) and was used as a pH value regulator. Bentonite (sodium form) was from Weifang, Shandong. Other reagents were used without further purification. Deionized water was used throughout all the experiments.

### 4.2. Methods

**Figure 10:**
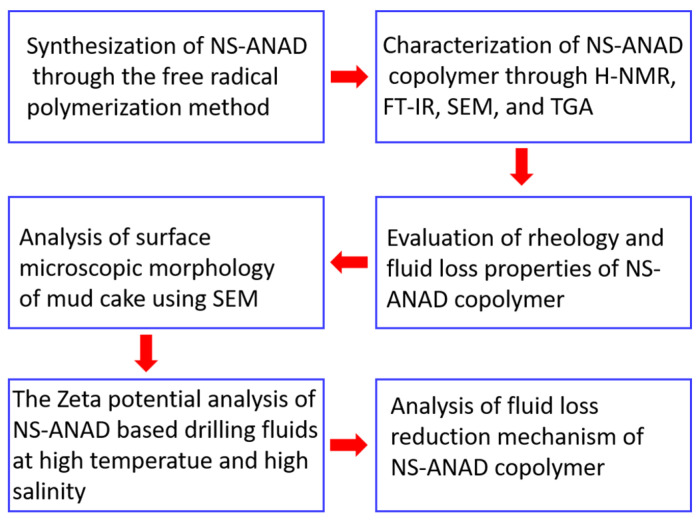
The workflow diagram of this research.

The following workflow diagram (Figure 10) presents the experimental steps for attaining the goal of this research.

#### 4.2.1. Synthesis of KH570/SiO_2_

First, 4 g of dried silica was ultrasonically dispersed in 60 mL of ethanol. Then, 0.8 g of the silane coupling agent, KH570, was dissolved in the alcohol/water dispersion, and the pH of the dispersion was adjusted to 4 with acetic acid. Next, the above 2 dispersions were mixed uniformly and stirred at 75 °C under reflux for 4 h. Finally, the reaction product was centrifuged, washed with ethanol several times, and dried under vacuum at 60 °C to obtain KH570/SiO_2_.

#### 4.2.2. Synthesis of NS-ANAD Co-Polymer

First, a certain mass of AM (2.13 g, 0.03 mol), NIPAM (6.78 g, 0.06 mol), AMPS (8.24 g, 0.04 mol), DMDAAC (3.22 g, 0.02 mol), and 0.41 g KH570-SiO2 (2 wt% of the total monomer mass) were dissolved in deionized water (the mass fraction of monomers was 20 wt%). The pH of the solution was adjusted to 7 with 20 mol/L of NaOH solution. Subsequently, the above solution was poured into a three-necked flask and stirred in a nitrogen atmosphere for 30 min to remove oxygen from the solution. Then, 0.2 g of APS was added to the solution that had been heated to 60 °C to initiate the polymerization reaction for 6 h. Finally, the reacted solution was purified with acetone to obtain an inorganic/organic co-polymer fluid loss reducer (NS-ANAD).

#### 4.2.3. Characterization

The ^1^H NMR test was conducted using the NMR spectrometer (Bruker AVANCE III NMR) with deuterated water (D_2_O) as the solvent. The Fourier transform infrared (FTIR) spectrometry (A225/Q Platinum ATR) of the sample was measured by the KBr tablet method (1 mg NS-ANAD mixed with100 mg KBr). The spectra in the wavenumber range of 4000−400 cm^−1^ were acquired at a resolution of 4 cm^−1^ with 16 scans. The thermal stability of the NS-ANAD was measured on a thermal analyzer (TGA, 1/1100 L, Mettler Toledo Co., Zurich, Switzerland) in an N_2_ atmosphere. The heating rate was 10 °C/min^−1^, and the temperature ranged from 40 to 600 °C. The micromorphology of the samples was observed by scanning electron microscopy (SEM, ZEISS, EVO-15, DE) and transmission electron microscopy (TEM, JEOL JEM2100F, Japan), respectively.

#### 4.2.4. Preparation of Drilling Fluids

In preparation for the base slurry, 16 g of bentonite and 0.48 g of Na_2_CO_3_ were slowly added into 400 mL of deionized water and stirred at high speed for 20 min, and then static standing at room temperature for 24 h to obtain a base slurry. Then, 0.5, 1.0, 1.5, and 2.0 wt% co-polymer NS-ANAD were added to the prepared base slurry and stirred at high speed for 20 min to obtain the NS-ANAD-based drilling fluids. Furthermore, 4, 15, 25, and 36 wt% NaCl were added to the 2.0 wt% NS-ANAD-based drilling fluid and stirred at high speed for 20 min to obtain the brine drilling fluids. The prepared drilling fluid was poured into an aging tank and heated in a roller oven through hot rolling at different temperatures (150, 180, 200, and 220 °C) for 16 h. After the hot rolling was completed, the aged drilling fluids were stirred at high speed for 30 min to measure the rheological and filtration properties.

#### 4.2.5. Performance of NS-ANAD in Drilling Fluids

The API filtrate performance was measured according to the American Petroleum Institute (API) standard, and the volume of fluid loss was recorded after 30 min under a pressure of 100 ± 5 psi. The rheological parameters of the drilling fluids were tested with a six-speed rotational viscometer (Qingdao Haitongda Special Instrument Co., Ltd., Qingdao, China) according to the American Petroleum Institute (API) standard. The formulas for calculating apparent viscosity (AV), plastic viscosity (PV), and yield point (YP) were as follows:

$$\mathrm{AV}=\frac{1}{2}\theta 600\;\left(\mathrm{mPa}\cdot s\right)$$

$$\mathrm{PV}=\theta 600-\theta 300\;\left(\mathrm{mPa}\cdot s\right)$$

$$\mathrm{YP}=\frac{1}{2}\left(\theta 300-\mathrm{PV}\right)\left(\mathrm{mPa}\cdot s\right)$$

#### 4.2.6. Fluid-Loss Control Mechanism Analysis

The mud cake formed after filtration was thoroughly dried at room temperature. The thickness of the mud cake was tested by a ZN-1-type instrument, and the micromorphology was analyzed by scanning electron microscope (SEM, ZEISS, EVO-15, DE). The Zeta potential of the drilling fluids was examined using a Zetasizer Nano ZS instrument (Malvern, UK), with the concentrations of drilling fluids diluted to 1.0 g/L.

## Data Availability

We don’t have any data.
